# Enhanced systematic delivery of fluconazole-loaded biotin-glutathione functionalized chitosan-g-proline carrier into the infected retinitis treatment

**DOI:** 10.1186/s12886-024-03305-z

**Published:** 2024-01-30

**Authors:** Qing Guo, Zheng Li, Fang Cao

**Affiliations:** 1 Ophthalmology, Department of Inner Mongolia Chaoju Eye Hospital, Hohhot Inner Mongolia, Hohhot, 010050 China; 2 Department of Ophthalmology, The 940 Hospital of PLA Joint Logistic Support Force, Lanzhou, Gansu 730050 China; 3 Department of Ophthalmology, Affiliated Chenzhou Hospital, The First School of Clinical Medicine, Southern Medical University, The First People’s Hospital of Chenzhou), Chenzhou, Hunan 423000 China

**Keywords:** Biotin, Chitosan, Fluconazole, Fungal infections, Proline, Retinopathy

## Abstract

**Background:**

The polymer-based facile and effective drug carrier approach was developed to treat superficial fungal infected retinopathy infections.

**Methods:**

Here, biotin-glutathione (B-GHS) functionalized with chitosan grafted proline (CS-g-P) moieties were fabricated with the loading of fluconazole (FLZ) for the treatment of retinopathy. FT-IR and XRD techniques were used to characterize chemical structural and phase changes of the prepared carriers The SEM results show that the sphere morphology with interconnection particle nature.

**Results:**

The particle diameter was found as ~ 6.5 and ~ 8.6 nm for CS-g-P/B-GHS and FLZ-loaded CS-g-P/B-GHS carriers, respectively. The negative surface charge was found as the values of CS-g-P/B-GHS and FLZ-loaded CS-g-P/B-GHS, such as -20.7 mV and − 32.2 mV, from zeta potential analysis. The in-vitro FLZ releases from the CS-g-P/B-GHS were investigated at pH 7.4 (PBS) as the tear fluid environment, and it was observed at 85.02% of FLZ release in 8 h reaction time. The sustained release was observed, leading to the necessity for prolonged therapeutic effects. The antifungal effect of the carrier was studied by the minimum inhibitory concentration (MIC) and the percentage inhibition of viable fungal count against *Candida albicans*, and it observed 81.02% of the zone of inhibition by the FLZ carrier.

**Conclusion:**

FLZ-loaded CS-g-P/B-GHS carrier could inhibit the biofilm formation in a concentration-dependent inhibition. Hence, A novel FLZ/B-GHS-CS-g-P carrier is a hopeful approach for effectively treating superficial fungal contaminations of the retina region.

## Background

Drug delivery and pharmacokinetics assume basic parts in creating contemporary retinal therapeutics of the new drug delivery system [1]. Retinal disease is expanding as a result of maturing, inherited, and diabetes [2, 3]. The appropriation of medicines to retinal targets is entreating and complicated because of unambiguous drug delivery in poor admirations [4]. Most accessible medicines and techniques arise in sub-remedial medication levels in the retina. An intravitreal infusion is the ebb and flow treatment approach for retinal illness [5]. The important downside of intravitreal infusion is that it causes torment due to expanded intraocular pressure. The infusion should be utilized frequently to upgrade drug focuses [6].

The polymer-based drug carrier system is improving the interaction of drugs in cells/tissue and the penetrability of drugs into cells [7, 8]. Biodegradable polymers are widely used in the synthesis of drug delivery systems with controlled and targeted drug delivery systems. These systems release the drug while the polymer is being swelled in the target site [9]. For effective drug delivery system preparation, chitosan has been utilized to prepare an admirable drug carrier system to cure various diseases [10]. The chitosan polymer is fundamentally utilized in drug carrier development improvements like disease, tuberculosis, and ophthalmic medication conveyance [11]. Chitosan is regularly made out of normal, nontoxic polymer to be viewed as greatly viable, biodegradable, and bioactive [12]. Tingting Xu et al. reported chitosan-glutathione-glycyl sarcosine (CG-GS) and layered double hydroxides (LDH) carrier systems for the treatment of mid-posterior diseases [13]. Glycylsarcosine (GS) is an active target ligand of the peptide transporter-1 (PepT-1), which could specifically interact with the PepT-1 on the cornea and guide the nanoparticles to the treating site.

The therapeutic potency, clearance, and bioavailability of the drug at the specific site of the ocular tissue include the carrier type and loading of drugs, etc. The targeted drug delivery system will minimize the side effects and enhance the therapeutic activity [14]. The associations between the transporter surface gatherings and the plasma surface membrane receptors represent the targetability and productivity of the take-up. Biotin (Vitamin B12) carries out a fundamental role in an entanglement of metabolic responses that are supplements for cell capabilities and improvement and keep up with development in the retinal cells. Biotin is a carrier across the blood-retinal hindrance, and it is utilized for retinal irregularity examples to defeat biotin lack [15, 16]. The ultimate objective of the current work is to create the chitosan-joined proline transporter framework for delivering fluconazole (FLZ) drugs. The great solvent nature and profoundly porous properties of FLZ make it a decent antifungal person as a BCS class-I particle [17]. We exhibit the amalgamation and assessment of medication conveyance framework to the conveyance of FLZ into the retinal as on ligand-receptor connections, subsequently working on restorative impact, controlled delivery, and medication focusing on nature.

## Methods

### Chemicals

Chitosan (CS) (medium molecular weight and 85% deacetylated), proline, biotin, glutathione, fluconazole, Sodium hydroxide, Potassium dihydrogen phosphate, 4-dimethylamino pyridine (DMAP), and Ethanol were purchased from Sigma Aldrich, China. *N*-Ethyl-*N*′-(3-dimethyl aminopropyl) carbodiimide hydrochloride (EDC.HCl), N-Hydroxy succinimide (NHS), Dimethylformamide (DMF), glutathione (GHS), and acetone were purchased from Sigma Aldrich, China. All the reagents and chemicals were utilized as such from the supplier with no additional purification. Double distilled (DD) water was used as a solvent for cleaning throughout the experiment.

### Preparation of chitosan grafted proline (CS-g-P)

Initially, 20 mg CS was prepared in 1% acetic acid in the ratio of 4 mg/1 mL at room temperature (RT, 27 °C), and then the pH of the CS solution was changed to 5.0 by using 1 M of NaOH solution. Then, the proline (10 mg) was dissolved in 0.01 M of pH 6.0 PBS (phosphate buffer solution) with stirring for 30 min at RT. The EDC.HCl and NHS were added to the proline solution with uniform stirring at 4 °C [18]. The CS solution dropwise was added into the proline reaction mixture under constant stirring at 4 °C and the stirring continued up to 24 h at RT. The reaction mixture was poured into the dialysis membrane (Molecular weight cutoff 12 kDa) and dialyzed for 3 days in deionized water, the excess EDC.HCl, NHS, and unreacted proline were removed, then the CS-g-P were freeze-dried at -40 °C and kept for further reaction.

### Biotin grafted glutathione preparation

Biotin grafted glutathione (B-GHS) coupling reaction was carried out through the standard amide bond formation generated bonds between biotin and glutathione was followed by the previously reported protocol. The EDC.HCl and NHS were used as the coupling agents [19]. The biotin (0.6 g) in 5 mL of DMF was treated with EDC.HCl (0.452 g) and NHS (0.257 g) for 15 min at 0 °C to activate the carboxylic group. The mixture was then stirred for 1 h at RT. The resultant solution was brought to pH 6.0 after 1 h with the addition of 1.0 M acetic acid/1.0 M NaOH solution and the stirring was continued up to 24 h. The GSH (1 g, 6 mmol) solution was prepared with 5 mL DMF solution and added in to the above mixture. The reaction mixture pH was maintained at pH 7.4. The precipitated product was filtered with cold diethyl ether and then washed with the same solvent. The solution was then lyophilized (Sub Zero Lyophilizer, China) at -40 °C for 8 h after being purified by dialysis (12,000 kDa molecular weight cutoff [MWCO], Himedia, China) against DD water for 48 h.

### Conjugation of CS-g-P and B-GHS

As per the previously described literature, the conjugation of B-GHS and CS-g-P polymer was carried out with a slightly modified procedure [20]. Briefly, the Dean-Stark apparatus was used for the ester bond formation, and a 1:1 molar ratio of CS-g-P (0.2 g) and B-GHS (0.2 g) was immersed in the DD water with the estimated amount of toluene in a 100 ml round bottom flask. The reaction mixture was then heated at 120 °C for 24 h to reflux with constant stirring and water removal before Sn(Oct)n (0.1 ml) was added dropwise. At 27 °C, the reaction mixture was cooled, and the precipitate was filtered through Whatman filter paper and vacuum-dried.

### FLZ loading in CS-g-P/B-GHS carrier

A stirring method carried out the loading of FLZ in the CS-g-P/B-GHS carrier. Briefly, 50 mg of CS-g-P/B-GHS and 10 mg of FLZ (10 mg) were mixed in 5 ml ethanol and it stirred up to 24 h at RT. After the stirring, the reaction mixture was centrifuged at 12,000 RPM for 20 min and dried particles were used for further investigation. Scheme 1 represents the schematic chemical reaction of the CS-g-P/B-GHS carrier synthesis.

### Fourier transform infra-red (FT-IR) analysis

FT-IR spectra were observed using a PerkinElmer Spectrum 100 series FT-IR spectrometer. The functional groups and modification of functional groups in the carrier were analyzed using the FT-IR spectrometer between 4000 − 400 cm^− 1^, while the resolution was ~ 4 cm^–1^ by accumulating 32 scans. The 10 gm of the sample was put in the mortar with an equal quantity of KBr pallet to make a tablet and the tablet was placed on an instrument holder for the FT-IR analysis.

### Zeta potential and particle size analysis

Zeta sizer Nano Series (Malvern, UK) equipment was used to measure the zeta potential using dispersed solutions of the samples at 27 °C. For the particle size analysis, the samples were diluted with 2 mL of DD water and illuminated by a laser beam. Then, the fluctuations of the scattered light were detected at a known scattering angle θ by a fast photon detector.

### SEM analysis

Scanning electron microscopy (SEM) was performed using SEM (TESCAN VEGA3 SBH) under high vacuum and ambient temperature with a beam voltage of 5 kV. Dilution of the sample dispersed solution was dropped onto glass plates for the SEM analysis and dried. The dry sample plate was then scanned on an SEM at 5 kV after being sputter-coated with Au powder (thickness 2 nm).

### Drug loading and releasing analysis

The FLZ-loaded CS-g-P/B-GHS carrier investigated the loading capacity through UV-1800, Shimadzu (Japan) at the wavelength of 265 and 287 nm. Briefly, the FLZ-loaded Cs-g/B-GHS carrier (10 mg) was vortexed for 10 min and centrifuged. The solution was analyzed in UV-Vis Spectrometer at λmax values of 265 and 287 nm. Similarly, the dialyzing membrane method was subjected to determine the release rate of FLZ from the FLZ-CS-g-P/B-GHS carrier. The FLZ release from the FLZ-loaded CS-g-P/B-GHS was carried out in the pH 7.4 phosphate buffer saline (PBS) solutions. The PBS solution was added to the dialysis bag with FLZ-CS-g-P/B-GHS carrier and it was stirred at 50 rpm, 27 °C. The 3 mL of the PBS solution was collected and replaced with fresh PBS at an interval of every 30 min period. The collected samples are tested in a UV-vis spectrometer at a wavelength of 265 and 287 nm. Loading capacity (%) and drug release (%) were determined by the below equations.

1 $$ \text{D}\text{r}\text{u}\text{g}\,\text{r}\text{e}\text{l}\text{e}\text{a}\text{s}\text{e} \left(\text{\%}\right)=\frac{\text{A}\text{b}\text{s}\text{o}\text{r}\text{b}\text{a}\text{n}\text{c}\text{e}\,\text{v}\text{a}\text{l}\text{u}\text{e}\,\text{o}\text{f} \,\text{r}\text{e}\text{l}\text{e}\text{a}\text{s}\text{i}\text{n}\text{g}\,\text{d}\text{r}\text{u}\text{g}}{ \text{A}\text{b}\text{s}\text{o}\text{r}\text{b}\text{a}\text{n}\text{c}\text{e}\,\text{v}\text{a}\text{l}\text{u}\text{e}\,\text{o}\text{f} \,\text{f}\text{r}\text{e}\text{e}\,\text{d}\text{r}\text{u}\text{g}}\times 100$$

2 $$ \text{L}\text{C}\left(\text{\%}\right)=\frac{\text{T}\text{h}\text{e}\,\text{t}\text{o}\text{t}\text{a}\text{l}\,\text{a}\text{m}\text{o}\text{u}\text{n}\text{t}\,\text{o}\text{f}\,\text{d}\text{r}\text{u}\text{g}-\text{F}\text{r}\text{e}\text{e}\,\text{a}\text{m}\text{o}\text{u}\text{n}\text{t}\,\text{o}\text{f}\,\text{d}\text{r}\text{u}\text{g}}{ \text{w}\text{e}\text{i}\text{g}\text{h}\text{t}\,\text{o}\text{f}\,\text{t}\text{h}\text{e}\,\text{d}\text{r}\text{i}\text{e}\text{d}\,\text{m}\text{i}\text{c}\text{e}\text{l}\text{l}\text{e}}\times 100$$

### Determination of zones of inhibition by agar diffusion method

Agar diffusion assay was performed to analyze the inhibition ability of FLZ-loaded CS-g-P/B-GHS carrier against *Candida albicans* (*C. albicans)* fungi. Pure FLZ drug was used as a standard antibiotic for comparison of the results. Two sets of four dilutions, each of CS-g-P, CS-g-P/B-GHS, and FLZ loaded CS-g-P/B-GHScarrier(50 µg/ml), were prepared in sterile Mc Cartney bottles. The carrier and standard drugs were dissolved in DMSO. To check for contamination, sterile nutrient agar plates were made and incubated at 37 °C for 24 h. The surface of the flooded plate, designated as quadrants on the back of the Petri dishes, was covered with four sterile filter paper discs (Whatman No. 1), each measuring 6 mm in diameter, which had been soaked in four different dilutions of the crude sample. Widths of the inhibition zone were determined in millimeters after the Petri dishes were incubated at 37 °C for 24 h. The pure FLZ drug was treated similarly, and the corresponding zone of diameters was compared.

### Determining the antibiofilm activity

The antibiofilm activity of usnic acid was assessed by the ring test at the concentrations of 25 µg/ml and 50 µg/ml, respectively. 1 ml of the PD broth (HiMedia) was supplemented with 20 µl of yeast cell suspension and usnic acid (25 µg/ml and 50 µg/ml) and incubated statically for 24 h at 37 °C and it followed previous literature [21]. The organism’s growth absorbance was read at 600 nm using a Multi-Label Reader (SpectraMax M3, USA). Then the planktonic cells were decanted, and the mature biofilm attached as a ring on the walls of the test tube was stained using crystal violet. A Leica TCS SP5 upright confocal laser scanning microscope (Leica Microsystems, Wetzlar, Germany) and an HCX APO L/0.90 W U-V-1 water immersion lens were used for image acquisition. Stains were excited with 488 and 633 nm laser lines and emissions were each detected with separate photomultipliers set to 505–535 nm and 620–670 nm respectively. Two z-stacks were acquired on each disc and fields of view were chosen at random (disc perimeter was avoided).

### Statistical analysis

ANOVA analysis was subjected to comparing the mean value of all the groups. *p* < 0.05 is measured as statistically significant. PRISM version 8.4.3 is used for all the statistical analyses.

## Results

### FT-IR analysis

**Fig. 1:**
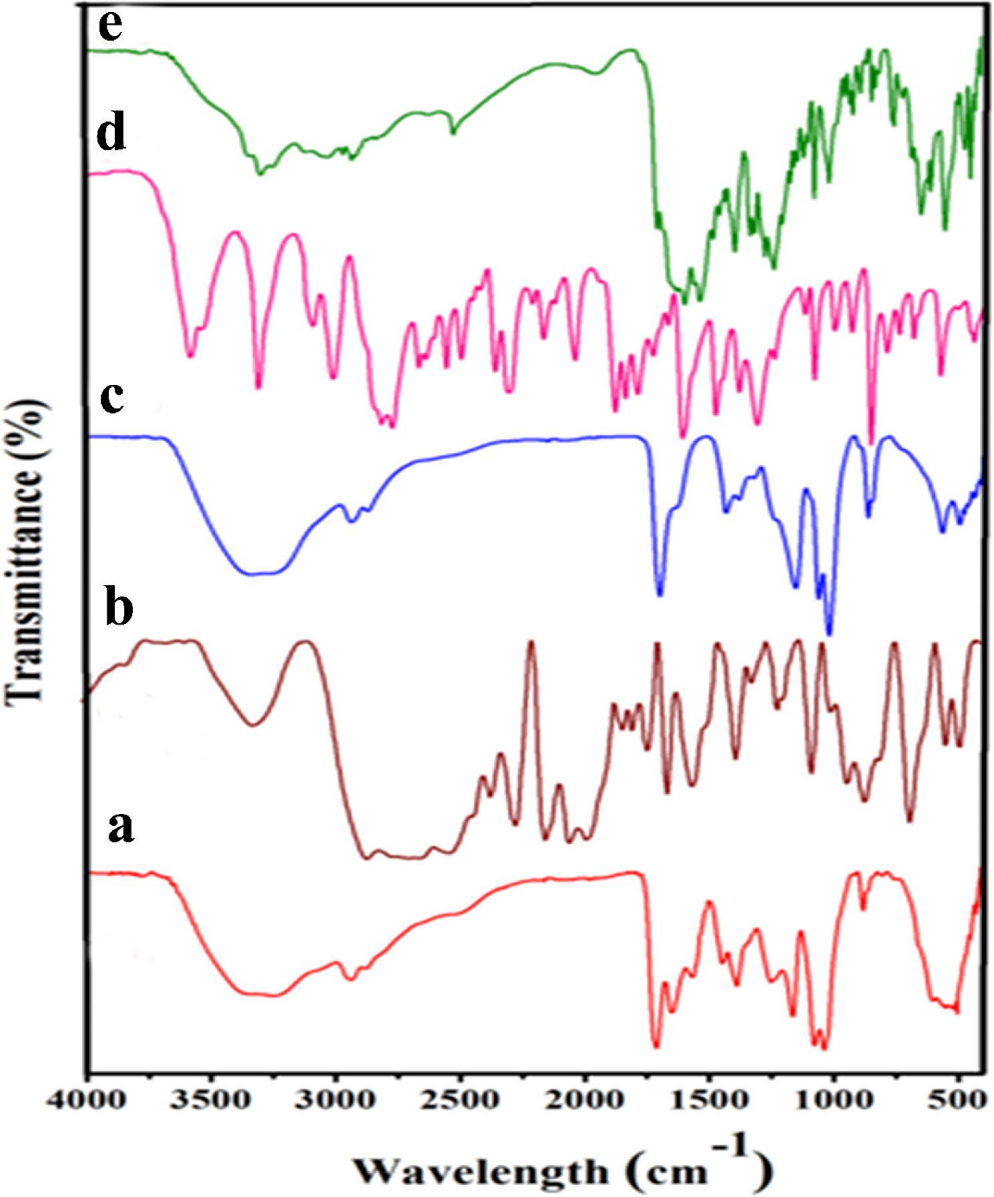
FT-IR spectrum of (**a**) CS-g-P, (**b**) B-GHS, (**c**) CS-g-P/ B-GHS, (**d**) FLZ drug, (**e**) FLZ-CS-g-P/ B-GHS

FT-IR studies were accomplished to confirm the synthesis of CS-g-P, B-GHS, CS-g-P/B-GHS, FLZ drug, and FLZ-loaded CS-g-P/B-GHS; the results are presented in Fig. 1. Figure 1a shows the typical spectra of CS-g-P, and it exhibits characteristic peaks at 1634 cm^− 1^ and 1539 cm^− 1^ matching the stretching and bending modes of the (C-O) and (-NH) of the amide (-CONH) group, which indicate the successful graft of proline in CS molecule [22]. The FT-IR spectrum of B-GHS shows the absorbance peaks at 1658 and 1554 cm^− 1^, respective to the -CO stretching and -NH bending modes of amide -CONH groups. The peaks at 543 cm^− 1^, 931 cm^− 1^, 1076 cm^− 1^, 1719 cm^− 1^, 2525 cm^− 1^, 3033 cm^− 1^, and 3366 cm^− 1^, which corresponds to the functional groups of the biotin and glutathione molecules by evidence from the previous reports [23, 24]. The peaks were due to the bond formation between the biotin and glutathione as biotinylated glutathione conjugation (Fig. 1b). The FT-IR spectrum of the CS-g-P/ B-GHS shows a highly intense peak arising at 1703 cm^− 1^, and it is corresponding to the C = O stretch, confirming esterification between B-GHS and CS-g-P moieties [25]. Also, the presence of other characteristic peaks 1719 cm^− 1^, 1076 cm^− 1^, and 931 cm^− 1^ is responsible for B-GHS, which resembles the confirmation of B-GHS functionalization in the CS-g-P moiety (Fig. 1c). FT-IR spectrum of fluconazole drug shows the vibrations of the different functional moieties present in the compound could be attributed to broad peak due to intramolecular hydrogen-bonded O-H stretching frequency in the range of 3600 − 2500 cm^− 1^ [26]. The 1619 and 1514 cm^− 1^ are due to the C = C stretch aromatic ring, and 1502 cm^− 1^ and 1420 cm^− 1^ are due to the triazole ring stretch. The triazole ring was breathing at 1138 cm^− 1^ and 1273 cm^− 1^ for the C-F stretch of the FLZ molecule (Fig. 1d) [27, 28]. The FLZ-loaded CS-g-P/ B-GHS attributed the maximum peak of FLZ retained, such as 1273 cm^− 1^, 1420 cm^− 1^, 967, and 846 cm^− 1^ peaks, which confirms that FLZ drug loaded in the CS-g-P/ B-GHS carrier (Fig. 1e).

### Carriers surface charge, size, and morphology

**Fig. 2:**
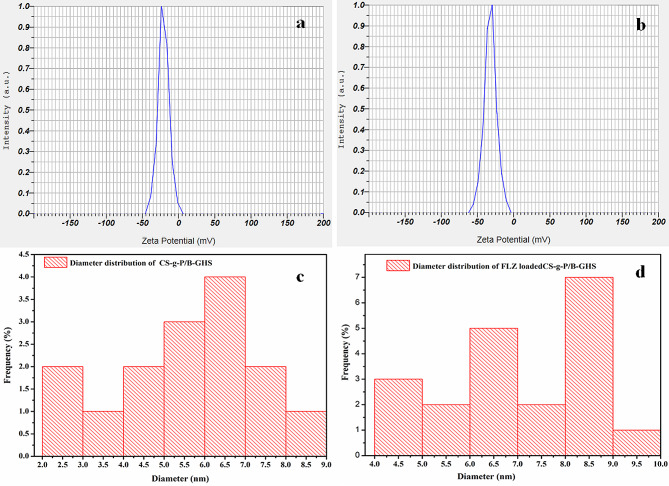
Zeta potential distribution (**a**) CS-g-P/ B-GHS and (**b**) FLZ loaded CS-g-P/ B-GHS carrier (**c**) Particle diameter distributions of CS-g-P/ B-GHS, and (**d**) FLZ loaded CS-g-P/ B-GHS carrier

**Fig. 3:**
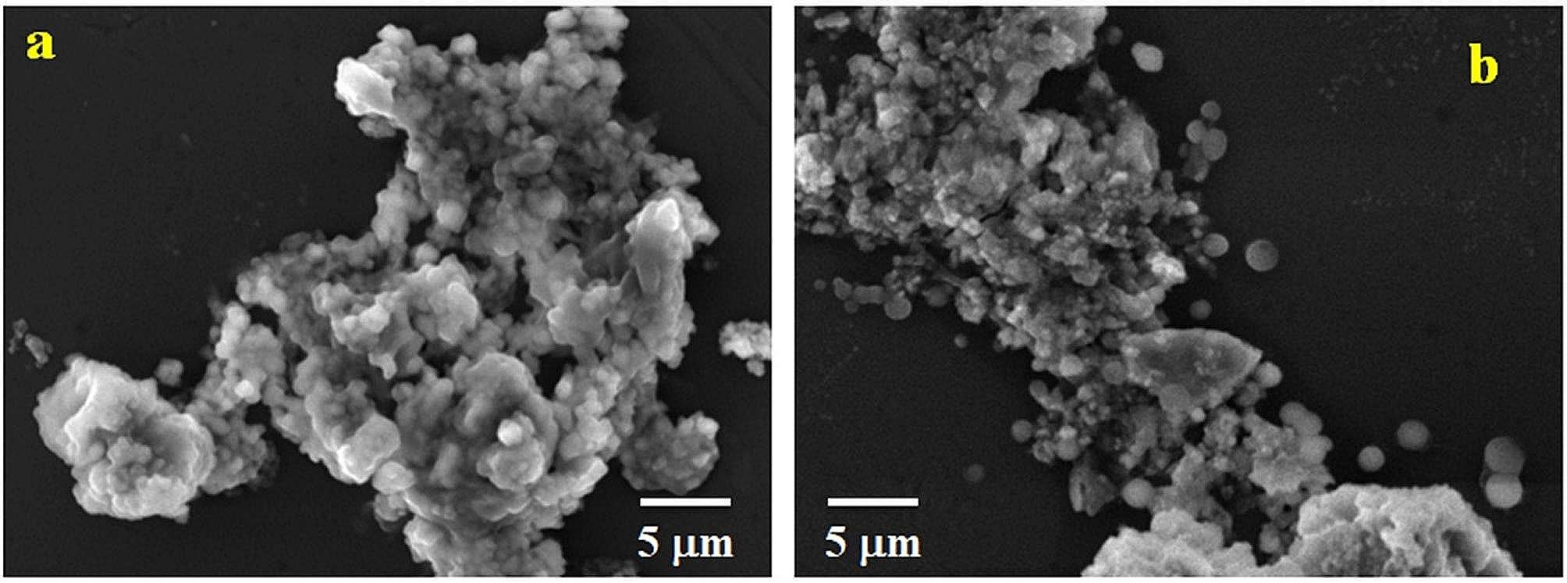
SEM images of CS-g-P/ B-GHS (**a**), and FLZ loaded CS-g-P/ B-GHS carrier (**b**)

The zeta potential values of CS-g-P/B-GHS and FLZ-loaded CS-g-P/B-GHS carriers are observed to be − 20.7 mV and − 32.2 mV (Fig. 2a and b). The negative surface charge esteem is fundamentally significant for restricting the positively charged surface of the cell layer. It could be a potential justification for the higher stability and expanded retinal penetrability [29]. Moreover, the typical measurement of the particles was noted as ~ 6.5 and ~ 8.6 nm of CS-g-P/B-GHS and FLZ loaded CS-g-P/B-GHS carriers, respectively (Fig. 2c and d). The morphology of the carrier surface was contemplated with SEM results, which denoted that the carrier system is round with a smooth surface and interconnected particle nature. The spherical shape of the carrier is reasonable for penetrability into the ocular system [30]. The SEM examination showed no distinctions between the CS-g-P/B-GHS and FLZ-loaded CS-g-P/B-GHS carriers regarding morphology (Fig. 3a and b).

### In-vitro release studies

**Fig. 4:**
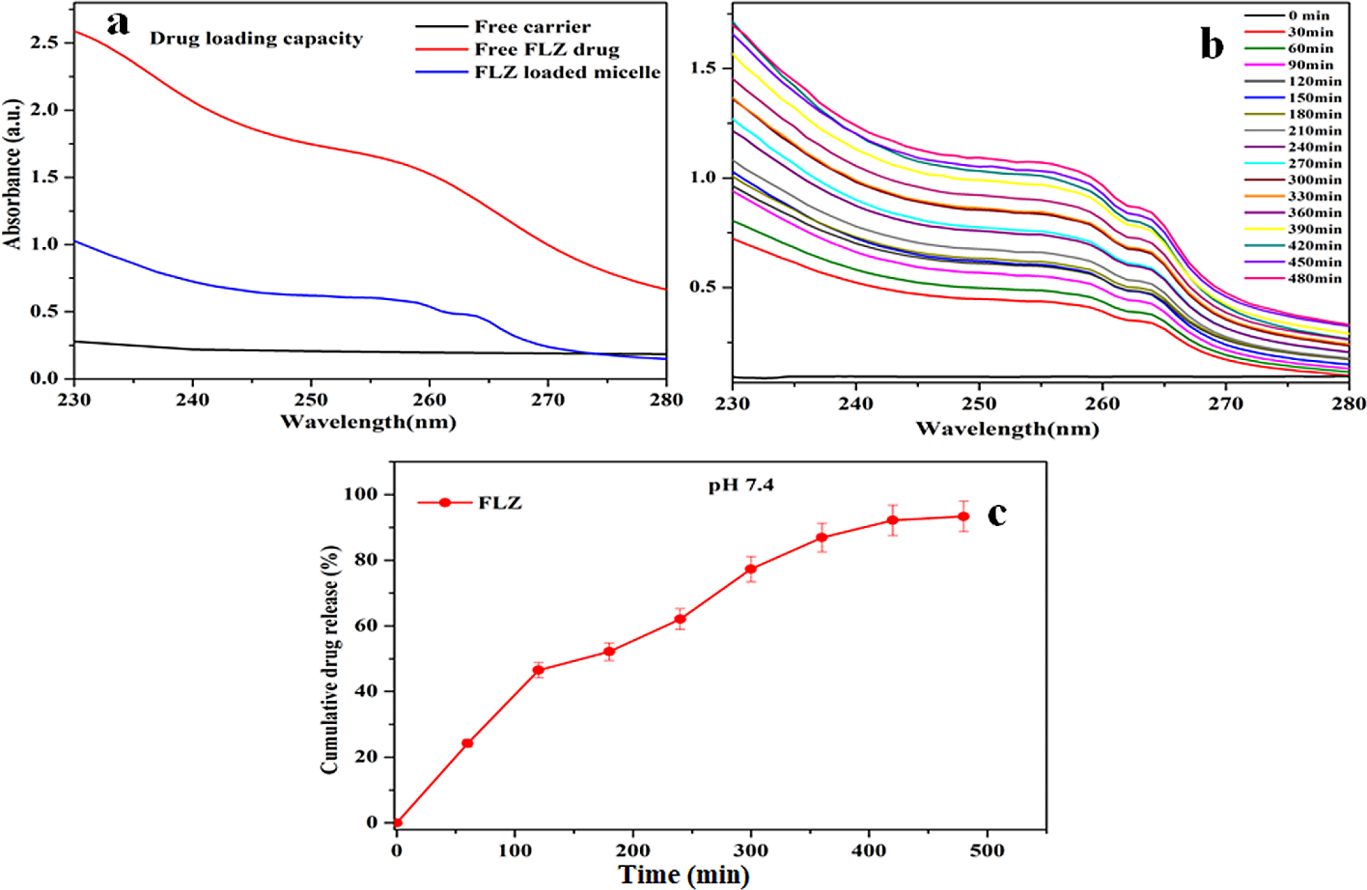
(**a**) FLZ loading capacity of CS-g-P/B-GHS, (**b**) In-vitro release of FLZ from FLZ/CS-g-P/B-GHScarrier at pH 7.4, and (**c**) Collective drug release pattern

The FLZ drug loading capacity of the CS-g-P/ B-GHS carrier results are given in Fig. 4a. The UV-visible absorbance intensity of the FLZ drug increased at the λmax value of 265 and 287 nm after the vortex. It indicates the high amount of FLZ drug loaded with 70.5% in the CS-g-P/B-GHS carrier. Generally, the drug delivery system has limited drug loading capacity and cost-effectiveness for drug administration [28]. While, the synthesized CS-g-P/B-GHS carrier has a good loading capacity of FLZ drug. FLZ-loaded CS-g-P/B-GHS carrier, the FLZ drug release was examined in a physiological tear environment (pH 7.4) at RT (27 ^◦^C). Figure 4b demonstrates the FLZ absorbance spectrum of FLZ from the CS-g-P/B-GHS carrier. It shows the controlled drug release based on the initial burst release obeys a controlled diffusion mechanism [31].

### Antifungal activity

**Fig. 5:**
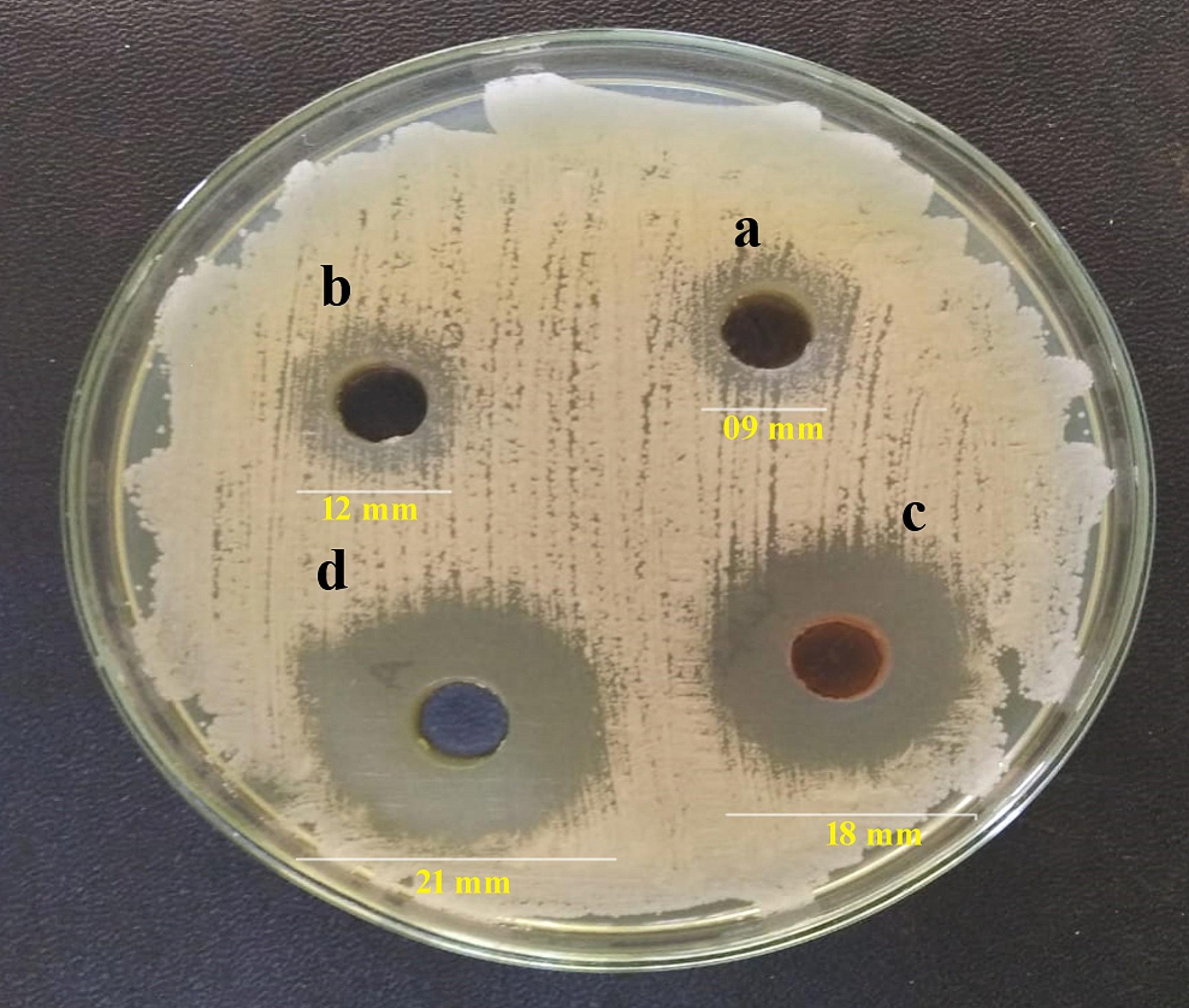
Antifungal activity of (**A**) CS-g-P, (**B**) CS-g-P/B-GHS, (**C**) FLZ drug, (**D**) FLZ loaded CS-g-P/B-GHS carrier

**Fig. 6:**
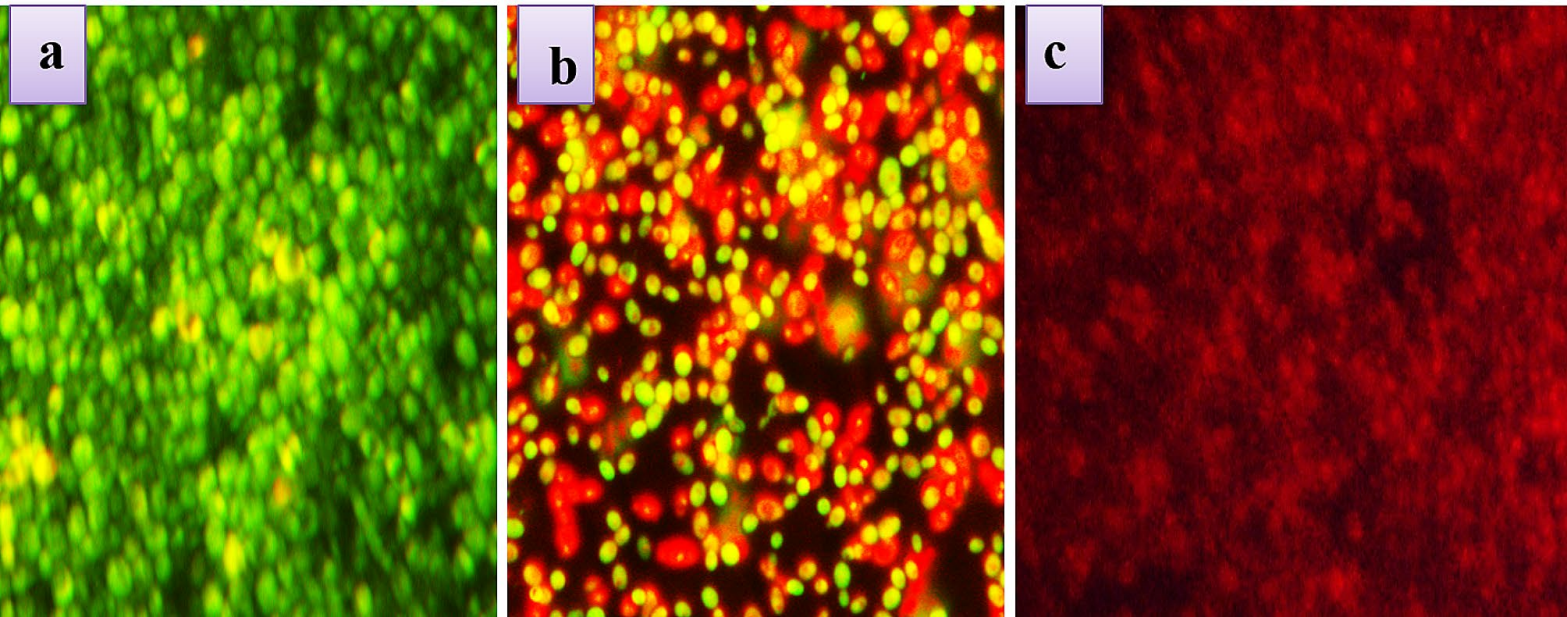
Biofilm inhibition by acridine orange staining by HCS analysis (**A**). *Candida Albicans* biofilm incubated with 1/2 MIC of (**B**) CS-g-P/B-GHS and (**C**) FLZ loaded CS-g-P/B-GHS carrier

FLZ-loaded CS-g-P/B-GHScarrier has significant antifungal activity in contradiction of *C. albicans* with a MIC value of 15.75 µg/mL. The highest percentage of the zone of inhibition was recorded against *C. albicans* (81.02%) at 1 mg/mL of FLZ-loaded CS-g-P/B-GHS carrier (Fig. 5). FLZ might enter *C. albicans* membrane and may not only act in the inside the fungus cell and extracellular site, but it also induced serious damage of cell membrane and cell wall, and consequently caused in the decreasing of protein production inside the cells [32]. Visualization of *C. albicans* biofilm by CLMS analysis and the results suggest that the FLZ-loaded CS-g-P/B-GHS carrier could inhibit the biofilm formation in a concentration-dependent inhibition (Fig. 6). Minimum inhibitory concentration (MIC) value of FLZ loaded CS-g-P/B-GHS carrier (15.75 µg/mL) completely inhibited the biofilm formation of *C. albicans.*

## Discussion

The synthesis of FLZ-loaded CS-g-P/B-GHS carrier was carried out by the series modification from chitosan as CS-g-P, B-GHS, CS-g-P/B-GHS and then FLZ drug was loaded. The prepared carrier system and its precursors were characterized by their physicochemical nature by FTIR, XRD, SEM, and TEM. The FLZ-loaded CS-g-P/ B-GHS attributed the maximum peak of FLZ retained, and it confirms that FLZ drug loaded in the CS-g-P/ B-GHS carrier. The typical measurement of the particles was noted as ~ 6.5 and ~ 8.6 nm of CS-g-P/B-GHS and FLZ loaded CS-g-P/B-GHS carriers and the SEM examination showed no distinctions between the CS-g-P/B-GHS and FLZ-loaded CS-g-P/B-GHS carriers regarding morphology. The size of the carrier to be adequately reasonable permits them to effortlessly saturate through the firmly pressed corneal epithelial cells and junctional buildings and defeat the corneal hindrance [33]. The drug’s slow release from the carrier could be accredited to the hydrophobic interactions between the drug molecules and the carriers’ hydrophobic region. The cumulative drug releases in PBS medium pH 7.4 were shown in Fig. 4c and observed at 82.0% in 8 h duration [34]. These results represent that CS-g-P/B-GHS has great potential applications for the distribution and sustainable release of FLZ in a tear environment. The main mechanism of antimicrobial activity due to FLZ may change the sterol profile of yeast by causing inhibition of ergosterol biosynthesis [35]. FLZ encourages lipid peroxidation, which may be one of the mechanisms involved in its Candida-cidal activity [36].

## Conclusion

The FLZ-loaded CS-g-P-B-GHS carrier was designed and prepared for the effective delivery of FLZ into retinal fungal infection. The spherical morphology with the interconnected particle nature was observed from the surface analysis of the carrier systems. Good stability with the surface charge of -32.2 mV and below 10 nm sizes of the carrier system was confirmed by the zeta potential and particle size analysis respectively. The antifungal activity of the FLZ-loaded CS-g-P-B-GHS carrier was observed at 81.02% against *C. albicans* at 1 mg/m. FLZ-loaded CS-g-P/B-GHS carrier has good biofilm inhibition ability against *C. albicans* with increasing concentration and increasing inhibition ability. Based on the results observed from the investigation, it can be concluded that fabricated retinol-targeted FLZ-loaded CS-g-P-B-GHS carriers systems can be used for the topical drug administration of ocular treatment of fungal keratitis and achieved to enhance the site-specific and sustainable release of the drug for the treatment of eye disease.

## Data Availability

All data generated or analyzed during this study are included in this published article.
